# Klotho and the Treatment of Human Malignancies

**DOI:** 10.3390/cancers12061665

**Published:** 2020-06-23

**Authors:** Aishani Sachdeva, Jerome Gouge, Christos Kontovounisios, Stella Nikolaou, Alan Ashworth, Kenneth Lim, Irene Chong

**Affiliations:** 1 The Royal Marsden NHS Foundation Trust, London SW6 6JJ, UK; aishani.sachdeva@icr.ac.uk (A.S.); c.kontovounisios@imperial.ac.uk (C.K.); 2 Department of Surgery and Cancer, Chelsea and Westminster Hospital, London SW10 9NH, UK; stella.nikolaou02@imperial.ac.uk; 3 Institute of Structural and Molecular Biology, Birkbeck College, London WC1E 7HX, UK; j.gouge@mail.cryst.bbk.ac.uk; 4 Helen Diller Family Comprehensive Cancer Center, University of California San Francisco, San Francisco, CA 94158, USA; Alan.Ashworth@ucsf.edu; 5 Division of Nephrology, Department of Medicine, Indiana University School of Medicine, Indianapolis, IN 46202-5181, USA; kjlim@iu.edu; 6 The Institute of Cancer Research, London SW3 6JB, UK

**Keywords:** Klotho, cancer, colorectal cancer

## Abstract

Klotho was first discovered as an anti-ageing protein linked to a number of age-related disease processes, including cardiovascular, renal, musculoskeletal, and neurodegenerative conditions. Emerging research has also demonstrated a potential therapeutic role for Klotho in cancer biology, which is perhaps unsurprising given that cancer and ageing share similar molecular hallmarks. In addition to functioning as a tumour suppressor in numerous solid tumours and haematological malignancies, Klotho represents a candidate therapeutic target for patients with these diseases, the majority of whom have limited treatment options. Here, we examine contemporary evidence evaluating the anti-neoplastic effects of Klotho and describe the modulation of downstream oncogenic signalling pathways, including Wnt/β-catenin, FGF, IGF1, PIK3K/AKT, TGFβ, and the Unfolded Protein Response. We also discuss possible approaches to developing therapeutic Klotho and consider technological advances that may facilitate the delivery of Klotho through gene therapy.

## 1. Introduction

The alpha-KLOTHO gene, named after the Goddess who spins the thread of life in Greek mythology, was first discovered as an anti-ageing gene in 1997 [1]. Kuro-o et al. demonstrated that mice with a deletion of approximately eight kilobases in the kl locus of the alpha-KLOTHO gene died prematurely at 8–9 weeks of age, and displayed phenotypes similar to advanced ageing, including arteriosclerosis, skin atrophy, osteoporosis, infertility, and emphysema. In contrast, transgenic mice overexpressing alpha-Klotho had an extended life span of up to 30% compared to wild type mice [1,2]. These discoveries sparked immense interest into the role of alpha-Klotho in ageing, and many studies have since linked alpha-Klotho deficiency or mutations, to a number of age-related disease processes, including cardiovascular, renal, musculoskeletal, and neurodegenerative conditions [3,4]. In addition, emerging research has also highlighted the potential role of alpha-Klotho in the pathogenesis of human malignancies [5,6,7]. Cancer is recognized to be an age-associated condition with the incidence of many cancers increasing with age [8]. Human malignancy is predicted to be the leading cause of death and the single most important obstacle to the increase of life expectancy in the 21^st^ century [9]. 

Cancer and ageing share comparable principles; the time-dependent accumulation of DNA damage is a contributing factor in ageing and also drives cancer progression [10,11,12]. DNA damage, alongside genomic instability, is an established hallmark of most cancers. This results in an increased frequency of mutagenesis during cell division [13,14,15] due to the malfunction of the genomic maintenance machinery and surveillance systems that usually drive damaged cells into either senescence or apoptosis [16,17,18]. Genetic defects driving genomic instability include loss of function mutations in caretaker genes encoding proteins responsible for detecting DNA damage, repairing damaged DNA, and preventing DNA damage [13,16,17,19,20,21,22], as well as gain of function mutations that activate downstream signalling pathways [14,15,23,24,25]. These pathways influence a number of capabilities acquired by cancer cells including the ability to evade apoptosis, cause tumour invasion and metastasis, enable cellular self-sufficiency, sustain angiogenesis, and facilitate unlimited cellular replicative potential [15]. Alpha-Klotho potentially influences these phenotypes through the inhibition of a number of signalling pathways such as insulin-like growth factor 1 receptor (IGF-1R), fibroblast growth factor (FGF), transforming growth factor β (TGFβ) and wingless-related integration site (WNT) [5,6,26]. 

**Figure 1:**
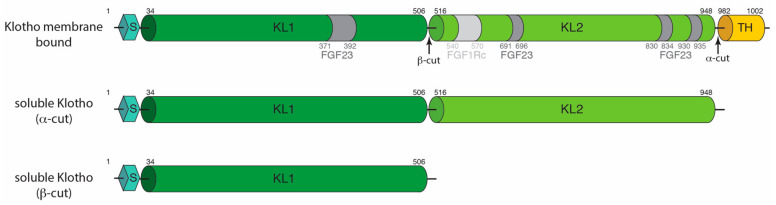
Domain representation of alpha-Klotho. The signal sequence (S), KL1, KL2, and the transmembrane helix (TH) are indicated on the diagram with residue numbering. The interaction with FGF23 and FGFR1c are indicated in the figure in dark and light grey, respectively. Soluble forms of alpha-Klotho are generated with the α- and β-cut.

Alpha-Klotho was originally identified in the distal convoluted tubules of the kidney in mice [27]. Since, the expression of alpha-Klotho has been seen in a variety of tissue types, including arterial, epithelial, endocrine, reproductive, and neuronal tissues in humans by targeted proteomic analysis in parallel with conventional antibody-based methods [28]. The human alpha-KLOTHO gene consists of five exons and four introns and ranges over 50 kb on chromosome 13q12 [29]. The gene encodes a type I single-pass 135 kDa membrane protein consisting of 1,012 amino acids [30,31]. It is composed of several domains, an N-terminal signal sequence, followed by two extracellular glycosidase domains (KL1 and KL2), a transmembrane helix and a short intracellular cytosolic domain of 10 amino acids at the C-terminus (Figure 1). Alpha-Klotho also exists in the circulation as both a soluble and secreted isoform [32]. Soluble Klotho (sKL) is generated from the cleavage of the full-length alpha-Klotho by membrane proteases (ADAM 10 and ADAM 17) in an α-cut to generate a 130 kDa protein that contains both the KL1 and KL2 domains but lacks the transmembrane helix and intracellular components [33] (Figure 1). Following the α-cut, the remaining transmembrane and cytosolic 5 kDa portion then undergoes proteolysis by γ-secretase [34]. A 65 kDa isoform is also generated by a β-cut and contains the KL1 domain. Secreted Klotho is generated by alternative mRNA splicing of alpha-Klotho [29], and its protein sequence is similar to the KL1 domain of alpha-Klotho, except for the C-terminus [29]. 

While alpha-Klotho and beta-Klotho are considered a family based on sequence similarity, the genes encoding these products are distinct and are located on different chromosomes; the alpha -KLOTHO gene is located on chromosome 13, and the beta-KLOTHO gene is located on chromosome 4 [29,35]. Beta-Klotho, localised to the cell membrane without a secreted form, is expressed principally in the liver and white adipose tissue [35,36] and has different functions to alpha-Klotho. The predominant functions of beta-Klotho involve bile acid synthesis, glucose uptake, and fatty acid metabolism [32]. Beta-Klotho forms a complex with both FGFR1c and FGFR4, thereby facilitating the activation of downstream signalling [37,38,39]. FGF21 binds to the beta-Klotho-FGFR1c complex, activates ERK1/2 phosphorylation and reduces bile acid synthesis [37,39,40,41]. FGF15/19 act through beta-Klotho-FGFR4 to suppress the synthesis of Cyp7a1, also leading to a reduction in bile synthesis in the liver [42]. For the purposes of this review, we will focus on the effect of alpha-Klotho (referred to as Klotho hereafter) in human malignancies, and discuss its therapeutic potential in the prevention and treatment of cancers.

## 2. The Tumour Suppressor Role of Klotho

The pleiotropic functions of Klotho have been increasingly recognised, including its importance in relation to tumorigenesis, cancer progression and prognosis [3,6,31]. In addition to being described as a tumour suppressor gene in numerous solid tumours and haematological malignancies, Klotho represents a possible therapeutic target for patients with these diseases, the majority of whom have limited treatment options [7,27,43] (Table 1). Klotho may play a role in inhibiting tumour initiation and progression. Several studies have evaluated protein expression in malignant tissue compared with matched normal tissue in different tumour groups. Klotho expression has been shown to be significantly down-regulated in malignant tissue compared to adjacent non-malignant tissue using immunohistochemical (IHC) techniques, including colorectal [44,45], pancreatic [46], gastric [47], oesophageal [48], breast [49], hepatocellular [50], ovarian [51], and renal cancers [52] (Table 1). Furthermore, more than ten single nucleotide polymorphisms (SNPs) have been identified in the human KLOTHO gene, and a number of studies have been performed to evaluate the associations between allelic variations in the KLOTHO gene and the aetiology of ageing-related diseases [53,54,55,56,57,58,59,60,61], including the effect of Klotho’s functional variants on cancer predisposition [62,63,64]. A meta-analysis of 29 case-control studies has been undertaken to derive a pooled estimate of effect and to quantify the variability observed between individual studies [65]. Relevant to cancer, the F allele of the F352V polymorphism was observed to protect against breast and ovarian cancer susceptibility. In a subgroup analysis stratified according to *BRCA1/2* mutation carrier status, the F352V polymorphism was associated with the overall cancer risk in *BRCA1* mutation carriers but not in *BRCA2* mutation carriers.

In contrast to the sizeable body of literature describing the loss of Klotho protein expression in human tumour tissues, there is a relative paucity in published data relating to circulating Klotho in solid tumours. In a study of 160 patients who underwent nephrectomy for renal cell carcinoma (RCC) [52], pre-surgery serum alpha-Klotho levels were lower in patients with more advanced disease; statistically significant differences were observed with tumour size (*p* = 0.003) and clinical stage (*p* = 0.0004). Further, a multivariate analysis showed that a low serum level of alpha-Klotho was an independent adverse prognostic factor for cancer-specific and progression-free survival in this study. Tang et al. measured serum Klotho levels in 40 patients with oesophageal squamous cell carcinoma (OSCC) and matched controls [48]. Despite the limitations in the size of this study, analysis from this dataset demonstrated significantly higher levels of serum Klotho in patients with OSCC compared with the control group (*p* < 0.001). However, a study undertaken by Pako et al. assessing plasma alpha-Klotho levels in 45 newly diagnosed lung cancer patients compared with 43 control subjects did not reveal any difference between the two groups [66]. Considering these data together, it is clear that further efforts are required to ascertain whether circulating Klotho has a role as a serum marker that could aid in the early diagnosis of different tumour types. Although circulating Klotho levels may not be altered compared with cancer tissues in all tumour contexts, functional data confirming tumour regression in several in vivo models that are not Klotho deficient supports further evaluation of Klotho as a candidate therapeutic target.

### 2.1. Breast Cancer

Rubinek et al. were one of the first groups to evaluate KLOTHO silencing in breast cancer [67]. By studying Klotho expression using IHC, the investigators found high Klotho protein expression in normal tissue samples compared with reduced expression in atypical ductal hyperplasia. Furthermore, KLOTHO promotor methylation was observed in five breast cancer cell lines as well as a proportion (8/23) of breast cancer samples, but not in normal breast samples, suggesting that loss of Klotho expression may be an early event in breast cancer development [67]. In a separate study, Wolf et al. used an antibody directed against the intracellular domain of membrane-bound Klotho to evaluate Klotho protein expression by IHC in a further 58 early-stage invasive ductal carcinoma samples, 47 pure ductal carcinoma in situ (DCIS) samples, and 11 normal breast samples [49]. Normal tissue adjacent to DCIS and invasive breast cancer was also analysed. They noted higher Klotho protein expression in all normal breast samples and in 19% of normal breast samples adjacent to invasive ductal carcinomas or DCIS, compared with only 17% in DCIS and 22% invasive ductal carcinoma (*p* < 0.0001). Furthermore, HA-tagged Klotho was overexpressed in MCF-7, and MDA-MB-231 breast cancer cells by transfection of pcDNA3 expression vector that resulted in reduced proliferation and a decrease in the number and size of surviving colonies by 84% and 72%, respectively. In another study focusing on structure-function analysis, Ligumsky et al. showed that overexpression of either Klotho or KL1, but not KL2, inhibited colony formation in breast cancer cells [68]. Moreover, KL1 administered in vivo was well tolerated and significantly slowed tumour formation in nude MDA-MB-231 breast cancer xenografts, illustrating differential activity of the Klotho domains that are relevant for potential therapeutic applications.

### 2.2. Colorectal Cancer

Through bioinformatics analyses of colorectal cancer TCGA datasets, Rubinstein et al. demonstrated reduced Klotho mRNA levels at all stages of disease compared to normal tissue. The analyses of publicly available DNA methylation datasets revealed a specific site in the first exon of KLOTHO, within a CpG island, that is hypermethylated in human colorectal cancer. Hypermethylation of the first exon, as well as promoter hypermethylation, negatively regulated Klotho expression in colorectal cancer [69]. This group demonstrated that overexpression using HA-tagged Klotho via transfection resulted in a reduction of surviving cancer colonies by at least 85% in colorectal HCT-116 and HT-29 cells. In addition, soluble KL1 protein inhibited the formation of colonies in the HCT-116 and SW489 human colorectal cancer cell line colonies. The investigators tested the effects of Klotho in vivo on either chemically induced carcinogenic, or orthotopic mouse models in which MC38 colorectal cancer cells were injected into the colonic submucosa. Mice treated with soluble Klotho exhibited significantly reduced weight and volume of the orthotopic tumour [69]. Furthermore, Klotho has been shown to be down-regulated through promoter methylation in 83.3% of colon cancer cell lines and 85% of primary colorectal cancer tissues [45]. Similarly, Li et al. demonstrated a reduction in tumour size and weight using a lentivirus-mediated Klotho vector, which was injected into subcutaneous tumours using a multi-site injection format, to evaluate the effect of Klotho on transplanted colorectal tumour xenograft models in immune-deficient mice [44].

### 2.3. Gastric and Oesophageal Cancers

From a clinical perspective, tissue expression of Klotho using IHC has been shown to inversely correlate with the histological grade and clinical stage of patients with oesophageal squamous cell cancer (OSCC) [48,50]. Importantly, the median survival time for patients with IHC-Klotho positive OSCC was observed to be 41.1 months compared with 24.9 months in patients with Klotho-negative OSCC (*p* < 0.01), emphasising the potential role of Klotho as a prognostic biomarker. In a functional study undertaken by Wang and colleagues, combined bisulphite restriction analysis revealed the presence of KLOTHO promoter hypermethylation in gastric cell cancer lines, including AGS, Kato III, MKN28, MKN45, NCI-N87, SNU1, and SNU16 [47]. Additionally, the evaluation of Klotho mRNA expression before and after exposure to the demethylating agent 5-aza-2′-deoxycytidine (5-Aza), revealed significant Klotho up-regulation, confirming the role of promoter hypermethylation in Klotho down-regulation. Although Klotho re-expression correlated with apoptosis and autophagy in this study, these results should be considered with caution, given the wide range of anti-metabolic activities and general toxicity associated with 5-Aza [47,70]. 

Micro RNAs (miRNAs) have been recognised to have tumour suppressor as well as oncogenic function depending on the genes they regulate [71]. He et al. found that levels of miR199a-5p were significantly upregulated in gastric cancer tissues compared with surrounding normal tissues (N = 103). They also observed that miR199a-5p could promote migration and invasion of gastric cancer cells. In addition, higher levels of miR199a-5p expression were found to be associated with a more advanced stage of disease, and that inhibition of miR199a-5p restored Klotho expression [72].

### 2.4. Pancreatic Cancers

Abramovitz et al. evaluated the expression and activity of Klotho in pancreatic cancer [73]. They undertook IHC analysis of Klotho protein expression in 18 pancreatic cancer tissues and found that 83% (15/18) displayed reduced Klotho expression compared with normal human kidney sections (*p* = 0.002). In Colo357, MiaPaCa2, and Panc1 pancreatic cancer cells, the investigators confirmed epigenetic silencing through promoter methylation of the KLOTHO gene. Further, pancreatic cancer cells transfected with an HA-tagged Klotho expression vector displayed a reduced number and size of surviving colonies compared with controls. Decreased cell viability was observed in Panc1 cells treated with soluble Klotho, and Klotho significantly inhibited anchorage-independent growth of these cells. In addition, pancreatic cancer mouse xenografts were treated with either Klotho, administered daily by intraperitoneal injection, or vehicle control; the xenograft weight and volume were significantly reduced in the experimental arm. Within the context of pancreatic ductal adenocarcinoma, high expression of miR-504 has been shown to correlate with low levels of Klotho, and miR-504 inhibition resulted in Klotho re-expression [46].

### 2.5. Other Tumour Types

Lee et al. examined epigenetic silencing of KLOTHO in human cervical carcinoma and found that loss of Klotho mRNA was seen in cervical cancer cell lines and invasive carcinoma samples but not in early, pre-invasive disease [74]. In this study, Klotho mRNA expression was restored after treatment with 5-Aza, and histone deacetylation was also found to be a notable epigenetic silencing mechanism that could be reversed using the histone deacetylase inhibitor, trichostatin A. In the liposarcoma model, Klotho overexpression was observed to significantly decrease cell proliferation and clonogenicity, as well as increase apoptosis induced by cytotoxic ER stressors such as gemcitabine, thapsigargin, and the Bcl-2 inhibitor ABT-737 [75]. Overexpression of Klotho in diffuse large B cell lymphoma (DLBCL) cell lines, LY1 and LY8, resulted in increased apoptosis and inhibition of proliferation [43]. Furthermore, treatment of melanoma cells with recombinant Klotho has been shown to decrease the development of metastases [49,76]. In a study evaluating the effects of Klotho overexpression in thyroid cancer, attenuation of proliferation and increased apoptotic activity were observed [77]. Tissue expression of Klotho using IHC has also been shown to inversely correlate with the histological grade and clinical stage of patients with hepatocellular carcinoma (HCC) [48,50]. Importantly, the overall survival of HCC patients with positive Klotho expression was significantly longer than those with negative Klotho expression. Inhibition of miR-10b has been shown to restore Klotho expression in models of non-small cell lung cancer [78]. Moreover, Klotho has also been shown to increase the efficacy of chemotherapy, including cisplatin in human lung cancer by modulating the phosphatidylinositol 3-kinase/protein kinase B PI3K/AKT pathway, indicating that Klotho therapy may be effective in combination with existing cancer therapies [79].

**Table 1:** Klotho is a tumour suppressor of human malignancies.

Tumour Type	Unmet Clinical Need	Model Systems/ Tissue Analysis
**Colorectal Cancer**	3rd most common cancer and 2nd leading cause of mortality [9]. Five-year survival for stage IV remains lower than 10% [80].	Cell lines HCT116, H-29, SW480, Colo-320 [69] Orthotopic C57BL/6 mice using MC38 colorectal cancer cells [69] Azoxymethane induced polyps in C57BL/6 immune-competent mice [44] Decreased Klotho expression in colon cancer tissues [45]
**Oesophageal and Gastric Cancer**	Oesophageal cancer is 7th most common cancer and 6th in mortality. Gastric cancer is the 5th most common cancer and 3rd leading cause of cancer death [9].	Cell lines MKN28, MKN-45, AGS, GC-7901, GES-1, NCI-N87, SNU1, SNU16 [7,47] Decreased Klotho expression in OSCC [48] Klotho expressing OSCC associated with improved survival [48]
**Pancreatic Cancer**	Survival rates have remained relatively unchanged since the 1960s [81], and is frequently diagnosed at a late stage [82].	Panc1, MiaPaCa2, and Colo357 [73] Female athymic nude mice Panc1 xenograft models [83] Decreased Klotho expression in Pancreatic cancer biopsies
**Breast Cancer**	Breast cancer accounts for 30% of new cancer diagnosis in women [82,84].	Cell lines MCF-12A, MCF-7, MDA-MB-231, MDA-MB-436, SKBR3, T47D, BT-474 [49] Decreased Klotho expression in breast cancer tissues [49,67] Nude mice xenografts using MDA-MB-231 cells [68]
**Lung Cancer**	Most common cancer diagnosed in both gender groups and the leading cause of mortality [9].	Cell line A549 [85]
**Ovarian Cancer**	The 6th most common cancer in females usually diagnosed late with high fatality rate [86].	Cell lines A2780, SKOV-3, OVCA 432, OVCAR-5, OVCAR-8, CaOV3, CaOV4 [51] C57BL/6 mice xenografts using A2780 cells [51] Decreased Klotho expression in invasive disease [51] Klotho high expressing tumours associated with improved survival [51]
**Thyroid Cancer**	Most common endocrine malignancy [9]. Frequently affects young women below the age of 30 years.	Cell lines FTC133 and FTC238 [77] Klotho inhibits STC1 expression in thyroid cancer cells [77]
**Melanoma**	Rising incidence [82]. 5th most commonly diagnosed cancer.	Cell lines G361, UACC903 and M93-047 [76] Cell lines characterised according to their levels of Wnt-5A [76] Klotho is lost as cells become more metastatic and may regulate Wnt-5A, which is correlated with more advanced metastatic disease. [76]
**Renal Cancer**	Rising incidence. Five-year survival for patients with renal cell cancer is less than 10% [87].	Human RCC cell line 786-O, OS-RC-2, ACHN, Caki-1 [52]. Klotho expression blunts RCC cellular migration and invasion in vitro [52]. Soluble, as well as tissue Klotho levels progressively reduced in patients with advanced clear cell renal cell carcinoma [52].
**Cervical Cancer**	Second leading cause of cancer death in women aged 20–39 years [82].	Cell line C-33A, CaSki, HeLa, SiHa, SNU-17, SNU-703, SNU-1160, SNU 1299 [74] Loss of Klotho mRNA observed in several cell lines and invasive carcinoma samples but not during the early, preinvasive phase of primary cervical cancer [74].

## 3. Pathways That Signal Downstream of Klotho and Their Impact on Cancer

**Figure 2:**
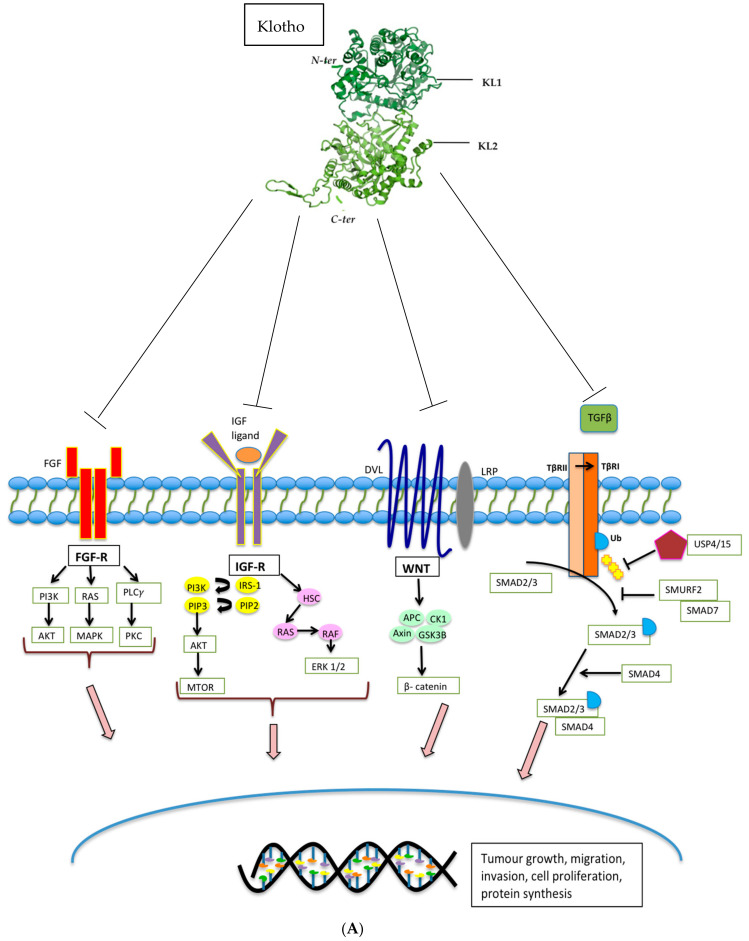

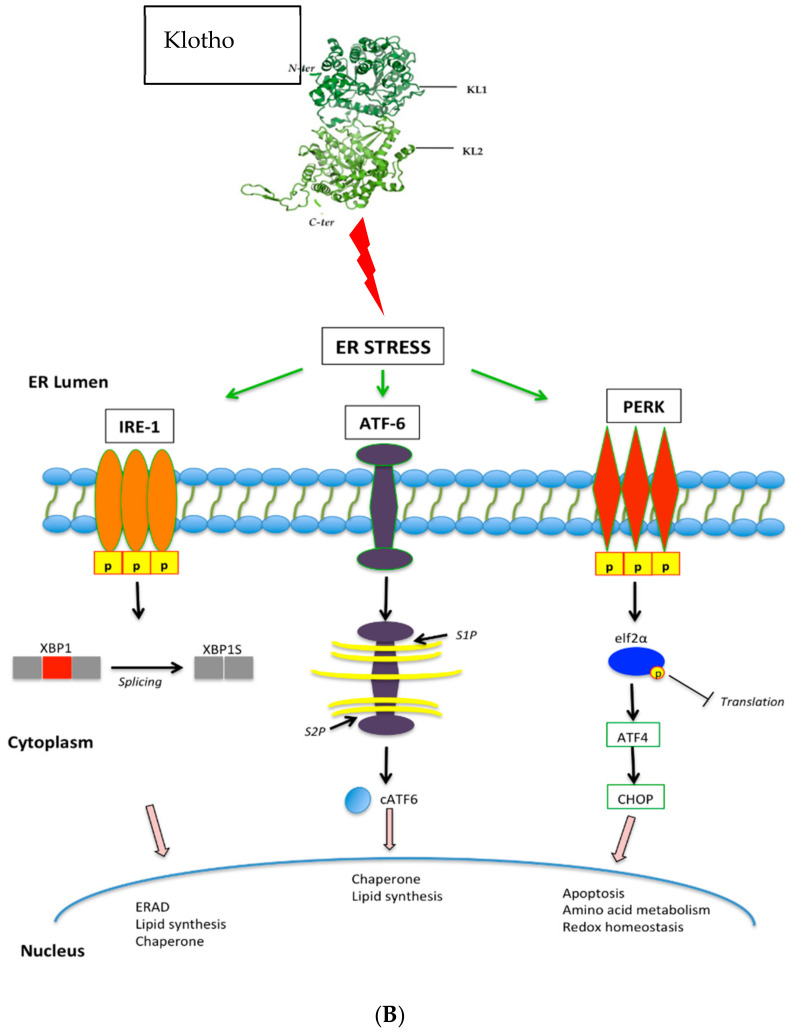

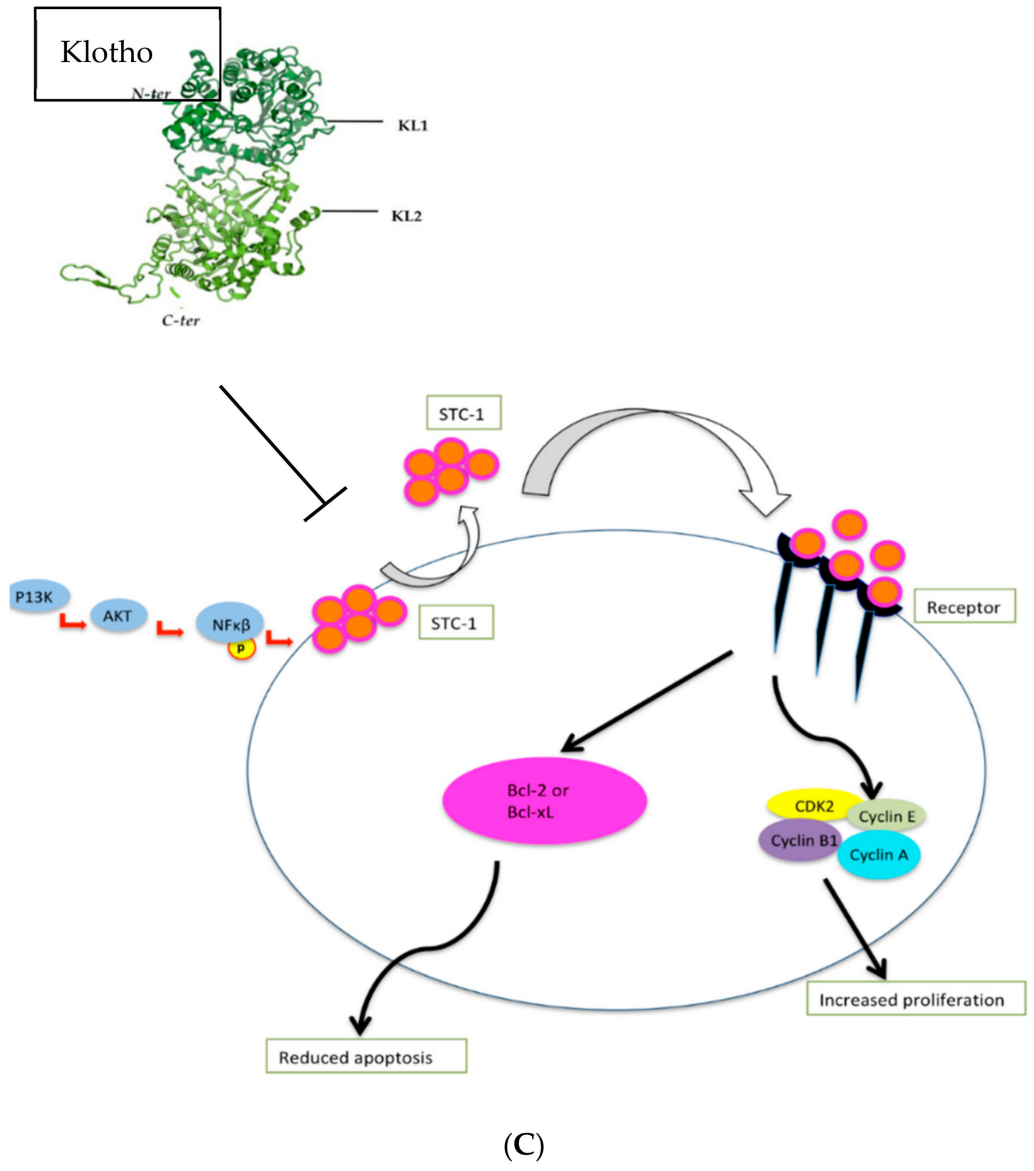
(**A**). Klotho inhibits FGF, IGF, WNT, and TGF-β signalling pathways, resulting in reduced tumour growth, tumour migration, tumour invasion, cell proliferation, and protein synthesis. (**B**) Klotho activates the Unfolded Protein Response, causing activation of PERK-elfF2α pathway and the subsequent increase in pro-apoptotic signals. (**C**). Klotho inhibits the Stanniocalcin pathway, resulting in decreased cell proliferation and increased cell apoptosis.

Soluble Klotho (sKL) has been shown to mediate the inhibition of a number of important pathways and regulate the activity of multiple proteins known to drive tumorigenesis, including insulin/ insulin-like growth factor 1 (IGF-1), Wnt-β-catenin, MAPK, PIK3A, AKT, Unfolded Protein Response (UPR), Stanniocalcin-1 (STC-1), and transforming growth factor-beta 1 (TGFβ) (Figure 2 and Table 2) [26,88]. However, the ligand binding sites of Klotho responsible for the downstream pathway modulation have not yet been fully characterised, and the mechanisms of action are not entirely understood.

### 3.1. The FGF Pathway

Through evaluation of the crystal structure of FGF complexes, Klotho was first found to function as a co-receptor for phosphatonin and FGF23. Together, these activate FGF receptor 1c (FGFR1c) in the kidney to stimulate phosphate wasting. Outside of phosphate regulation, the FGF pathway is also involved in diverse biological events such as cell differentiation, migration and proliferation, and Klotho may play a central role in modulating these pathways [89] (Figure 2a). Wolf et al. studied the effects of Klotho on the activation of the FGF pathway by transfecting breast cancer cells with a Klotho expression vector followed by exposure to bFGF. Using phosphorylation of ERK1/2 as an indicator for activation of the FGF pathway, the investigators noted stimulation of this pathway in the Klotho transfected breast cancer cells, with inhibition of cellular proliferation [49]. In contrast, a study focused on pancreatic cancer models found that Klotho overexpression or treatment with sKL reduced the growth of pancreatic cancer cell lines and xenografts, and inhibited FGF pathway signalling, resulting in reduced cell proliferation [73]. These data indicate that the effects of Klotho on FGF signalling may well be context-dependent.

### 3.2. The IGF-1R Pathway

IGF-1R is a receptor tyrosine kinase that stimulates proliferation by regulating downstream targets, including IRS proteins, the PI3K-AKT-TOR and RAF-mitogen-activated protein kinase (RAF-MAPK) pathways [90] (Figure 2a). High levels of circulating IGF-1 have been shown to be associated with an increased risk of several common cancers [90,91]. Klotho has been shown to have an impact on insulin physiology through inhibition of insulin and IGF-1 phosphorylation resulting in reduced activation of downstream targets and inhibition of intracellular signalling [2]. Studying breast cancer, Wolf et al. investigated the effects of expressing Klotho in MCF-7 breast cancer cells in which IGF-1R is highly expressed. Reduced phosphorylation of IGF-1R, as well as downstream targets, including IRS-1, AKT1, GSK3β, and ERK1, was observed in Klotho transfected cells following exposure to IGF-1 [49]. Similar results were seen in the pancreatic cancer cell lines, Panc1, MiaPaCa2, and Colo357 [73]. The IGF-1 pathway can also drive cellular proliferation in haematological malignancies; a study using LY1 and LY8 human DLBCL cells transduced with a Klotho expressing lentivirus, revealed that increased proliferation was observed in cells that were subsequently treated with IGF-1 compared with those that were not. In addition, reduced tumour growth and decreased cellular proliferation (Ki67) was seen in DLBCL Klotho over-expressing xenograft models, as well as xenografts treated with recombinant human Klotho. Taken together, these results support the tumour suppressor function of Klotho and its modulation of IGF-1R signalling [43]. Delcroix et al. undertook an important study investigating whether Klotho impacts the aggressiveness of liposarcomas (LPS), a tumour type in which IGF-1R signalling is frequently upregulated [75]. Through the analysis of publicly available data, significant downregulation of Klotho mRNA expression in 61 dedifferentiated liposarcomas (DDLPS) compared with 49 adipose tissue controls was detected. Amongst patients with LPS, lower expression was found to be associated with poorer prognosis. To assess the functional role of Klotho in high-grade LPS, a DDLPS cell line stably expressing Klotho was established and used to demonstrate reduced ERK1/2 phosphorylation compared with the control cell line, even during IGF-1 stimulation. Klotho overexpression was also observed to abrogate an IGF-1-induced Ca^2+^ response and significantly decrease cell proliferation and clonogenicity. In addition, increased apoptosis induced by cytotoxic endoplasmic reticulum (ER) stressors such as gemcitabine, thapsigargin, and ABT-737 was observed. The authors also demonstrated that the afore-described cellular effects could be counteracted by IGF-1R-dependent signalling and activate Ca^2+^-dependent ER stress. Furthermore, the mechanism by which Klotho caused cell death in this LPS model was shown to be related to increased reticular Ca^2+^-leakage.

### 3.3. The PI3K/Akt Pathway

The PI3K/Akt is an important downstream component of the IGF-1R pathway that regulates gene transcription, cell cycle events, and cell proliferation [92] (Figure 2a). In a study undertaken by Li et al. SW480 and HT29 colon cancer cell lines were used to evaluate the mechanism by which Klotho suppresses the growth and invasion of colon cancer via the PI3K/Akt pathway. In colon cancer cells transduced with a Klotho lentiviral construct, the investigators showed that Klotho downregulates the expression of IGF-1, PI3K, and AKT (mRNA and protein), with subsequent inhibition of growth and invasion of colon cancer cells [44]. In a different study relating to renal cancer, Klotho expression was inhibited using shRNA; this resulted in increased PI3K/Akt protein expression. Klotho, PI3K, and AKT protein expression was evaluated in 125 renal carcinoma specimens, and this revealed a negative correlation between Klotho and PI3K/Akt expression. Additionally, relative to early renal cancer tissues, advanced renal carcinoma tissues showed lower expression of Klotho and higher levels of PI3K, indicating a negative correlation [93]. In gastric cancer models, Klotho has also been shown to inhibit MAPK pathway phosphorylation, which signals downstream of IGF-1R [47,70].

Moreover, Dalton et al. undertook a study to better understand the mechanism by which sKL acts to mediate IGF-1 driven PI3K/Akt signalling [31]. Using HeLa cells, the authors demonstrated that sKL binds lipid rafts, highly dynamic microdomains that are rich in cholesterol and sphingolipid. These lipid rafts compartmentalise cellular processes such as signal transduction and membrane trafficking. They identify α2-3-sialyllactose, present in the glycan of monosialogangliosides that cluster in lipid rafts, as sKL targets, and show that sKL binding to lipid rafts alters lipid organisation and down-regulates raft-dependent IGF1-stimulated PI3K/Akt signalling in preference to glycosylated proteins and isolated gangliosides in non-raft membranes. Taken together, this study defines Klotho as a physiological regulator of lipid raft structure and function that may mediate its pleiotropic functions.

### 3.4. The WNT Pathway

Aberrant activation of the WNT signalling pathway is associated with the development of numerous human cancers, including colorectal and HCC [94]. This highly conserved pathway governs developmental processes, including cell proliferation and differentiation, with β-catenin being the critical effector in the canonical pathway [7,94] (Figure 2a). The frequent inactivation in colorectal, gastric, and breast cancer of soluble WNT antagonists like the frizzle related proteins (SFRP) is known to activate the canonical WNT pathway [95,96,97]. Aberrant WNT signalling is also recognised to be an early progression event in up to 90% of colorectal cancer cases [95] and is considered a potential therapeutic target where inhibition might slow or halt the development of cancer [98]. However, the effective clinical implementation and delivery have not yet been achieved largely due to treatment toxicity [99]. The Klotho protein is a secreted antagonist of the WNT signalling pathway; using HEK-293 cells transfected with a WNT luciferase reporter assay, Liu et al. found that secreted Klotho effectively inhibited WNT activity. Furthermore, using a Klotho- deficient mouse model of accelerated ageing, they discovered that various tissues from young Klotho-deficient mice exhibited stem cell depletion, increased progenitor cell senescence as well as increased WNT signalling [100]. They went on to show that the mechanism of Klotho-mediated WNT inhibition was as a result of Klotho binding to WNT ligands, namely Wnt3A and Wnt5A; thereby impeding binding of these ligands to their cell surface receptor [6,94]. In melanoma samples, a negative correlation of Wnt5A and Klotho expression has been observed. Using melanoma cell lines, Camilli et al. showed that Klotho suppressed Wnt5a internalisation and hindered the cleavage of Filamin A [76]. In colorectal cancer models, Klotho overexpression has been shown to reduce overall β-catenin expression, inhibiting transcriptional pathway activity by binding to the Wnt3a ligand, and thereby decreasing nuclear translocation of β-catenin [69]. The *APC* gene, which functions as a negative regulator of β-catenin is mutated in up to 85% of sporadic colon cancers, emphasising the importance of targeting the WNT pathway in this tumour type [101]. Finally, in primary HCC models, Klotho expression induced apoptosis by negatively regulating the WNT/β-catenin pathway [50,102].

### 3.5. The TGF-β Pathway

TGF-β signalling plays a biphasic role in the development and progression of cancer [103,104,105]. In normal cells and early stages of cancer progression, TGFβ acts as a tumour suppressor [106] with the ability to induce apoptosis [107,108,109] and inhibit cell cycle progression by triggering G1 cell cycle arrest [107,110]. However, in the late stages of cancers, including melanomas, gliomas, breast, and colorectal cancers, cells can become resistant to the tumour-suppressive effects of TGF-β by developing somatic loss of function mutations and acquiring activating mutations within alternative oncogenic pathways [111]. Despite this, cancer cells have the ability to remain responsive to TGF-β signalling, thus promoting epithelial-to-mesenchymal transition (EMT), invasion and metastasis [112,113]. Within the tumour microenvironment (TME), cancer-associated fibroblasts (CAFs) secrete TGF-β and interleukin 11 (IL-11), an inducer of TGF-β that activates the oncogenic STAT3 pathway [114]. TGF-β can also enable the differentiation of stromal mesenchymal stem cells (MSCs) into myofibroblasts that stimulate tumour growth by producing extracellular matrix and growth factors [115], as well as enhance anti-tumour immunity [116]. Doi et al. undertook a study to explore the interaction between secreted Klotho and TGF-β signalling [26]; using cultured renal epithelial cells with TGF-β1 in the presence or absence of secreted Klotho protein, they observed that Klotho inhibited TGF-β-induced phosphorylation of SMAD2 in a dose-dependent manner. They identified direct protein-protein interaction between Klotho and TGF-βR2 cell surface receptor, thereby explaining the ability of Klotho to inhibit TGF-β signalling. Further, overexpression of TGF-βR2 restored TGF-β-induced activation, confirming that Klotho acts to abrogate TGF-βR2 signalling. In addition to proving that Klotho replacement therapy suppresses renal fibrosis by preventing TGF-β from binding to TGF-βR2, the investigators also confirmed that the secreted Klotho protein inhibited TGF-β signalling and suppressed epithelial-to-mesenchymal transition (EMT) in lung adenocarcinoma cells. Klotho inhibited TGF-β induced phosphorylation of SMAD3, reduced binding of TGF-β to the cell surface, and attenuated TGF-β-induced EMT responses. Furthermore, they also showed that Klotho administration suppressed the metastases of lung cancer xenograft models. Taken together, this data provides evidence that Klotho has the ability to inhibit TGF-β-induced EMT responses by directly interacting with the TGF-βR2 cell surface receptor. sKL has also been shown to abolish the fibrinogenic effects of TGFβ [117,118].

### 3.6. The Unfolded Protein Response and Stanniocalcin Pathways

The UPR is a complex of signalling pathways that are able to maintain a productive endoplasmic reticulum (ER) protein-folding environment (Figure 2b). These pathways can be triggered by low oxygen levels or a restriction in nutrients and have been identified as playing a vital role in the development of various cancers [119]. The main components of the UPR pathway comprise the eukaryotic translation initiation factor 2 -alpha-kinase 3 (PERK), the endoplasmic reticulum to nucleus signalling 1 (IRE1), and activating transcription factor 6 (ATF6). The activation of PERK leads to the phosphorylation of other factors, which inhibit protein synthesis, regulation of detoxifying enzyme expression, and activation of downstream pro-apoptotic genes. Rubinstein et al. assessed the activation of the UPR pathway by Klotho in colorectal cancer cells by measuring the levels of spliced XBP1 mRNA, a commonly used marker for the activation of the UPR system. Not only was XBP1 found to be elevated in response to Klotho, exposure to a UPR inhibitor, tauroursedeoxycholic acid (TUDCA), partially rescued the effect of Klotho on colorectal cancer cell colony formation, supporting the ability of Klotho to modulate UPR pathways [69].

Stanniocalcin (STC), a secreted glycoprotein hormone involved in calcium and phosphate homeostasis [120] that is overexpressed in colorectal, hepatocellular, non-small cell lung cancer, and thyroid cancer [121], is also regulated by Klotho [122] (Figure 2c). Using human thyroid cancer cell lines, Dai et al. examined the relationship between Klotho levels and expression of STC isoforms, STC1 and STC2, and found that STC-1 mRNA and protein expression was significantly lower in Klotho-overexpressing cells, and that STC-1 silencing induced apoptosis whilst inhibiting cellular proliferation [77].

## 4. Therapeutic Approaches to Klotho Delivery

Despite the considerable interest in Klotho since its discovery over two decades ago, no Klotho-based therapies have reached clinical trials for a variety of reasons. Several strategies focused on delivering, restoring, or modulating Klotho levels have been considered. Delivery of the recombinant full-length Klotho protein due to its inherent large molecular weight and potential instability is challenging. One reasonable approach would be to consider information derived from the available structures of Klotho [123,124,125]. Overall, therapeutic interventions have been limited due to the biology of Klotho still being relatively poorly understood [118].

The identification of molecules that could modulate Klotho levels or Klotho-dependent signalling has been of interest, particularly for the treatment of neurodegenerative conditions or neurological malignancies where the introduction of an exogenous Klotho protein into the brain is impractical due to its large molecular weight and inability to cross the blood-brain barrier [126]. Moreover, due to the as yet unknown identity of the Klotho receptor, the development of molecules that can function as Klotho mimetics is not currently a viable strategy. Recently, several small molecules have been identified by high-throughput screening that can induce endogenous Klotho mRNA and protein expression [126]. Using the KL promoter to drive the expression of luciferase, high-throughput screening was conducted to identify compounds that elevate luciferase expression by at least 30%. Hit compounds were further evaluated in vitro by incubation with opossum kidney and Z310 rat choroid plexus cells, and these compounds led to elevated Klotho levels that were found to be physiologically active [127].

Gene therapy, defined as the introduction of genetic material into a target cell for therapeutic benefit, represents an alternative approach to treating disease [128]. To date, more than 2,000 clinical trials employing gene transfer have taken place, and the majority of gene therapy clinical trials have targeted cancer [129,130,131,132]. Although the concept of gene therapy appears theoretically simple, this approach has encountered significant hurdles when attempting to achieve optimal delivery and expression in target cells [133]. Historically, unwanted effects have included immunogenicity associated with adenoviral vectors, insertional mutagenesis, variation in viral tropism, the off-target activity resulting in adverse clinical outcomes ranging from mild flu-like symptoms to the death of participants within clinical trials [134]. However, with new technological advances in gene delivery and editing methods, as well as a number of viral-based drugs entering the clinic, there is the potential to deliver a ‘one-time’ treatment option without corrupting the genome that is safe, efficient, and specific [128,133]. In particular, adeno-associated virus (AAV) vectors have shown increasing promise due to their gene delivery efficacy, lack of pathogenicity, and strong safety profile [135]. AAV is a single-stranded DNA parvovirus with a 4.7 kb genome composed of the rep and cap genes flanked by inverted terminal repeats (ITRs) [136,137]. Recombinant AAV vectors can be generated by replacing the endogenous rep and cap genes with an expression cassette consisting of a promoter driving a transgene of interest and sequences causing the addition of a poly (A) tail [135]. Furthermore, the control of AAV-delivered transgene expression in vivo is possible through the engineering of reversible RNA on-switches, which may improve the safety and efficacy of gene therapies and broaden their use [138]. Whilst the modulation of Klotho expression in transgenic mice has been shown to extend lifespan by up to 30%, it is increasingly recognised that prevention or treatment of these diseases may involve the perturbation of multiple genetic pathways [2,139,140]. Taking this in to consideration, Davidsohn and colleagues undertook an in vivo study to evaluate gene therapies based on three longevity associated genes (fibroblast growth factor 21 [FGF21], Klotho, the soluble form of the mouse transforming growth factor receptor 2 [sTGFβR2]) delivered individually and in combination using AAV, and explored their ability to alleviate four age-related diseases—obesity, type II diabetes, heart failure, and renal failure [141]. They observed that a single formulation combining two separate gene therapies into a single agent was able to treat all four diseases. Whilst this data may be context-dependent and the investigators did not use cancer models, these results illustrate the promise of gene therapy for treating diverse age-related diseases and exhibit the potential of combination gene therapy that may improve longevity by addressing multiple diseases at once.

DNA methylation is a covalent modification of chromatin that is critical for regulating biological processes such as cellular differentiation and maintenance of cellular homeostasis [142,143,144,145,146]. Aberrant DNA methylation has been observed in numerous tumour types, neurodegenerative diseases, and genes related to ageing, including KLOTHO [147,148,149]. Epigenetic silencing of KLOTHO by hypermethylation is well established and, in theory, disruption of methylation could prevent Klotho loss. Bearing this in mind, Wang et al. showed that pharmaceutical demethylation with 5-aza resulted in the upregulation of Klotho [47]. The results of this study support efforts to edit the methylation state of specific loci; recent advances in CRISPR-Cas9 technologies allow improved flexibility and specificity, modulated by single-guide RNA [150], facilitating active demethylation by employing the ten-eleven translocation (TET) family of methylcytosine dioxygenases [151,152,153,154,155,156,157,158,159]. These technological developments address ways in which methylation can be reversed and have the potential to provide a therapeutic advantage in tumours that display loss of Klotho protein expression.

## 5. Conclusions and Future Perspectives

Klotho was initially discovered as an anti-ageing protein but has since generated considerable interest due to its potential therapeutic role in cancer biology, which is perhaps unsurprising given that cancer and ageing share similar molecular hallmarks. Studies comparing normal and tumour tissues have illustrated that the downregulation of Klotho is associated with cancer development and worsening of patient outcomes. This has highlighted Klotho’s potential role as a tumour suppressor, as well as a prognostic tumour biomarker with the potential to aid the early detection of malignancies. In addition, Klotho over-expression generally leads to decreased cancer cell viability, and treatment with soluble Klotho has resulted in reduced tumour volume in pre-clinical cancer models, including stomach, pancreas, colon, and breast. However, there are challenges that lie ahead in the advancement of Klotho as a therapeutic target. First, the exact mechanism by which Klotho exerts its anti-tumour effects is poorly understood and likely to be context-dependent. The abrogation of the diverse biological pathways that signal downstream of Klotho have been shown in several cancer models, as discussed here. However, the ligand-binding domains of Klotho responsible for downstream pathway modulation are not fully characterised, and the observation that Klotho binds to monosialogangliosides to regulate membrane microdomains opens up the possibility that pathway alterations via these gangliosides may be the critical link in deciphering the anti-neoplastic effects of Klotho [118]. Secondly, a satisfactory method of delivering therapeutic Klotho remains to be identified. For example, the potential instability and large molecular weight of Klotho makes the delivery of recombinant protein challenging as a therapeutic agent. Alternative approaches have been to identify small molecule agonists that modulate levels of Klotho [126] with the possibility of re-purposing already licensed drugs that are ready to be evaluated within future clinical trials. However, it is uncertain whether these compounds alone are able to elevate Klotho levels to those required for therapeutic benefit, especially when contemplating patient comorbidities, such as renal dysfunction, which would impair intrinsic Klotho production. Although technological advances in gene therapy have been made with regard to developing suitable vectors for efficient delivery and protein expression in target cells, the clinical translation of this approach within human trials is far from straight forward. In conclusion, research in Klotho is accelerating, not only within the renal and cardiovascular space but also in the area of oncology where new therapeutic approaches are urgently required.

## Figures and Tables

**Table 2:** Pathways that signal downstream of Klotho.

Pathway	Tumour Type	Assay
WNT/β-catenin	Colorectal, Oesophageal, Pancreatic, Hepatocellular, Bladder, Uterine cervix, Melanoma	TOP/pFOPFLASH luciferase assay to measure TCF-mediated transcription. Western blot to assess β-catenin expression following Klotho overexpression mRNA Klotho expression (RT-PCR) inversely correlated with β-catenin expression
FGF23	Breast, Pancreatic, Ovarian, DLBCL	Klotho overexpressing cells treated with FGF/FGF23. Phosphorylation of ERK1/ 2 western blotting used as an indicator for activation of FGF pathway
IGF-1R	Breast, Pancreatic, Ovarian, DLBCL	Phospho-IGF1R expression assessed by western blotting in Klotho overexpressed cells. CCKB assay assessing the effect of Klotho overexpression of IGF1 induced cell proliferation
PI3K/Akt	Colorectal and breast	Klotho overexpressing cells showed a significant decrease of p-PI3K and p-AKT expression by western blotting
UPR	Colorectal	Affymetrix gene expression screen RT-PCR
TGF-β	Lung	Identified direct protein-protein interaction between Klotho and TGF-βR2 cell surface receptor Klotho inhibited TGF-β induced phosphorylation of SMAD3, reduced binding of TGF-β to the cell surface and attenuated TGF-β-induced EMT responses in A549 cells
